# How will marine plastic pollution affect bacterial primary producers?

**DOI:** 10.1038/s42003-020-0789-4

**Published:** 2020-02-05

**Authors:** Sasha G. Tetu, Indrani Sarker, Lisa R. Moore

**Affiliations:** Department of Molecular Sciences, Macquarie University, Sydney, NSW 2109 Australia

**Keywords:** Ecology, Microbiology

## Abstract

We demonstrated in our recent *Communications Biology* paper how marine photosynthetic bacteria, *Prochlorococcus*, are adversely affected by leachates from commonly used plastics. This study was one of the first to consider how substances leaching from plastics may affect marine primary producers and demonstrated that plastic pollution has the potential to negatively impact a wider range of organisms than previously appreciated. We outline here key outstanding questions regarding how ocean plastic pollution may impact small, but essential, marine microbes and discuss how these can be addressed.

## Abstract (web summary)

Following up on their recent study in Communications Biology Sasha Tetu et al discuss how plastic pollution of the oceans may affect marine microbes as well as strategies to identify the substances responsible for leachate toxicity and to further understand their impact.

## Plastic pollution affects creatures great and small

**Fig. 1:**
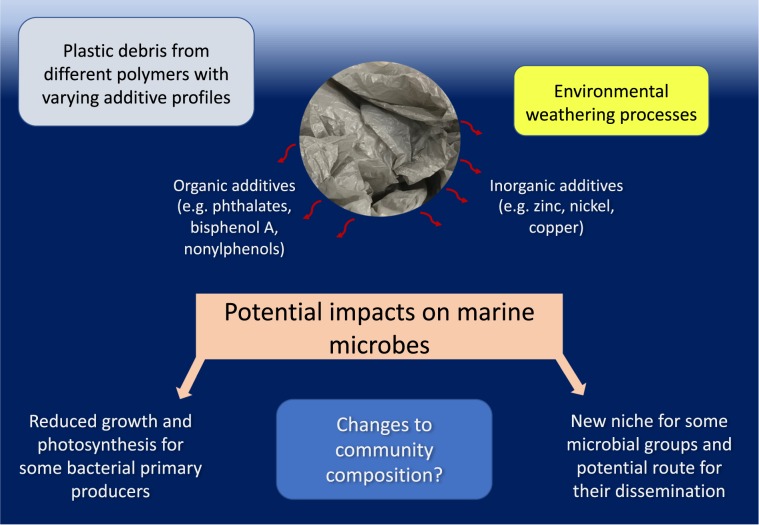
Current knowledge and key outstanding questions regarding the potential impacts of plastic pollution on marine bacteria.

It is now well recognised that marine plastic pollution threatens fish, bird and mammal species around the world. Images of animals entangled in plastic debris or stomachs filled with plastic pieces have helped galvanise scientific and public interest in this critical, worldwide issue. However, relatively few studies have considered how marine plastic pollution may affect the smallest life in our oceans, marine microbes (Fig. 1). Such microorganisms are critical to the marine food web, photosynthetic primary production and biogeochemical cycling.

When marine plastic pollution research has considered microorganisms, the focus has largely been on looking at what colonises and biodegrades plastic debris (reviewed recently by Jacquin et al.^1^). From these types of studies is appears that certain groups of bacteria will tolerate, and perhaps even benefit from marine plastics debris. Such findings are important and investigating bacteria with degradative capabilities may eventually result in improved technologies and processes for dealing with plastic waste^2^. However, focusing predominantly on these aspects can result in overly simplified appraisals of how bacteria may be affected by marine plastics. In a recent review that evaluated ecosystem impacts of marine plastics on different biota, bacterial diversity and/or abundance was assessed as being positively impacted by plastics, with this assessment scored as having good confidence^3^. Where the composition of bacterial communities colonising plastics have been examined, however, there is considerable variability in how well different bacterial families colonise common polymer types^4^. It is also clear that the bacterial taxa which predominate in biofilms on plastic in marine waters are distinctly different to those residing in the surrounding seawater^5^, indicating that planktonic marine bacterial lineages are not the main groups benefiting from plastics as a new potential niche.

The negative impact of marine plastics has largely been linked to plastic ingestion or entanglement, which marine bacteria are too small to suffer from, but this is not the only way by which plastic pollution may affect marine biota. In addition to providing a surface to colonize, plastics can also leach a variety of compounds. Plastics may provide some dissolved organic carbon sources for some bacteria to metabolise^6,7^ but they can also leach organic compounds and metals that negatively impact growth of a variety of microorganisms, including marine larvae^8,9^ and algae^10^. Most plastic items are manufactured using a variety of additives, such as UV stabilisers, plasticizers, metals, dyes and flame retardants^11^. These substances which are not chemically bound to the polymer can leach from plastic items and end up in the environment, an issue which is now the subject of increased investigation (reviewed in ref. ^12^). In our *Communications Biology* paper, we looked to expand our understanding of the breadth of possible marine plastic impacts by investigating how *Prochlorococcus*, an abundant, ecologically important bacterial primary producer, is affected by exposure to leachates from common plastics^13^.

## Plastics and *Prochlorococcus* don’t mix

In many regions of the world’s oceans *Prochlorococcus* are present in vast numbers, carrying out photosynthesis and oxygen production on an impressive scale. *Prochlorococcus* is perhaps the most plentiful photosynthetic organism on Earth, with an estimated yearly abundance of around 3 octillion (~10^27^) individuals^14^. These tiny, abundant bacterial primary producers help support the marine food chain and contribute substantially to global carbon fixation and oxygen production^15^.

In open ocean environments where *Prochlorococcus* are most numerous, plastic pollution is now recognised as a potential cause for concern^16^. To determine whether the chemicals leaching from such plastics might impact bacteria, as well as larger sea life we investigated the impact of plastic leachate on two different groups of *Prochlorococcus* that are found at different depths within the surface lit layers of the oceans. What we found was that both groups were unable to grow, photosynthesise or produce oxygen when high levels of leachates from two common plastics were added to their artificial seawater growth media^13^.

When cells were exposed to lower amounts of leachate, the two different *Prochlorococcus* strains differed in their sensitivity. This suggests some *Prochlorococcus* groups, and likely other bacteria, may vary in their capacity to tolerate exposure to plastic pollution. Thus, there may be “winners and losers” in ocean regions which accumulate high levels of plastic debris.

To tease apart potential reasons for the strain-specific responses to leachate exposure, our study also used whole genome RNA sequencing to look at gene transcription-level responses. Each strain was observed to respond to plastic leachate exposure with differential transcription of a substantially different suite of genes. One common feature in the response, however, was the reduction in the transcription of genes encoding key enzymes involved with photosynthetic carbon fixation. This, together with the observed reduction in photosynthetic oxygen production at sub-lethal leachate levels, suggests that photosynthesis in some marine bacteria may be particularly susceptible to the effects of plastic leachate exposure.

## Considering the community

Moving forward, it will be important to look at the effects of plastic-associated toxicants at the community level. Considerable work has been done with respect to microbial community composition within the “plastisphere”^17^, but to our knowledge there are no published studies examining the effects of plastic leachates on seawater microbial communities. In addition to expanding the set of organisms for which there is information on plastic leachate responsivity, community level analyses are important as the response of specific microbes may be influenced by the composition of the wider community. For instance, if some microbes are able to metabolise some of the leached chemicals^18^, then the more sensitive community members may be able to survive exposure to plastic leachates that may have otherwise impacted their fitness. Additionally, if sensitive microbes die quickly, then new bioavailable organic matter may provide an additional food source, stimulating growth of less sensitive microbes and resulting in indirect changes in community structure. Microcosm experiments have been used previously to investigate the impact of other pollutants, such as herbicides and polycyclic aromatic hydrocarbons, on marine phytoplankton communities^19,20^. We are using a similar approach to investigate the effect of plastic leachates on marine microbial communities from coastal and open ocean waters in order to determine how members of mixed communities respond in terms of photosynthetic capacity, population growth and changes in community structure.

## Do weathered plastics retain their toxicity?

To better understand how plastic waste in the environment is likely to affect marine bacteria we must also begin to consider how weathering affects leachate toxicity. Plastics may take hundreds to thousands of years to degrade in the environment^21^, however little is known regarding leaching dynamics and potential toxicity over months or years, let alone longer time frames. Almost all of the studies which have investigated leachate toxicity have focused on the effects of newly purchased, unused plastics as a first step in investigating potential impacts, including our initial study. One exception is the work of Bejgarn et al.^22^, which compared toxicity of new plastics with equivalent materials which had been artificially weathered, using the copepod *Nitocra sinipes*. This work reported that toxicity of some tested polymers was altered by simulated weathering (UV irradiation), with toxicity increasing for some products following irradiation, whereas other materials showed a decrease or no change in their toxicity. These findings highlight the potential for environmental exposure to alter toxicity and the need to investigate how estuarine or marine weathering processes may affect leachate toxicity. Recent work by Kedzierski and colleagues analysed the desorption and absorption phenomena for three different plastic types over more than a year of immersion in marine waters and examined changes in cytotoxicity of plastics during this period of weathering^23^. They reported both desorption and adsorption of different organic and inorganic substances, including some endocrine disruptors, over this time, with results varying for different plastic items and polymer types^23^. We are now looking at how estuarine weathering of plastic items affects leachate toxicity to gain a clearer picture of how environmental plastic pollution impacts may be altered by the degree of weathering.

## Can we identify the substances responsible for leachate toxicity?

Our present understanding of the potential biotic impacts of plastic leachates would also benefit from identification of the specific substances within leachates that are contributing to toxicity. Early ecotoxicological investigations into leachate toxicity found that different plastic items varied considerably in their toxicity, even when the base polymer was the same^24^. This is likely due to the complexity and variability in the precise set of additives which go into the manufacture of different items. Toxicity Identification Evaluation (TIE) testing methods can be applied to gain information on the likely classes of toxicants^24^. However, identifying the precise inorganic and/or organic components that contribute to toxicity requires follow-up investigations. This can be particularly problematic with organic components, as leachate from a single item may contain very large numbers of different organic substances and their breakdown products, many of which are difficult to precisely identify.

From our initial study, we were able to see that HDPE bags and PVC matting leached a complex mixture of organic compounds, with PVC containing a more diverse set of components, with tentative identification only possible for a very small fraction of these. Both plastics also leached inorganic metals, with zinc observed at particularly high levels, especially in the PVC product. Zinc is a common component of heat stabilisers, fillers and colourants used in plastic manufacture, and has been reported as an important component in previous leachate studies, particularly those involving PVC materials^8,10^. Leachate components such as zinc, which are abundant and widely used in plastic manufacture, are potential targets for future studies focusing on how specific additives contribute to toxicity to various biota. Such studies have been undertaken on other specific known plastic additives, such as recent work investigating the toxicity of fluorescent additives to microalgae^25^. Efforts to determine which chemical additives contribute substantially to toxicity may help facilitate development of plastic products that leach fewer or lower concentrations of toxic substances.

## Conclusions

Analytical advances in microbiology and molecular biology have helped to highlight the importance of microorganisms in all global processes including the cycling of carbon and oxygen production. Considering key microbes, such as photosynthetic marine bacteria, in efforts to monitor and assess environmental pollutants will help provide a more comprehensive picture of the ecosystem level impacts of such stressors. Our study on *Prochlorococcus* was a first step in this direction and subsequent studies involving a wider range of microorganisms, as well as considering microbial communities are now needed. Addressing the problem of marine plastic pollution requires research across many fields: from biological investigations into the breadth of biotic impacts and chemical analyses of weathering processes through to modelling the sources and movement of plastic pollution and engineering solutions for improved manufacturing and better handling of plastic waste. Understanding how plastic pollution affects marine bacteria is an important component of such efforts to predict and mitigate the impact of plastic pollution on our oceans.
